# Facing a Second Wave from a Regional View: Spatial Patterns of COVID-19 as a Key Determinant for Public Health and Geoprevention Plans

**DOI:** 10.3390/ijerph17228468

**Published:** 2020-11-16

**Authors:** Olga De Cos, Valentín Castillo, David Cantarero

**Affiliations:** 1 Department of Geography, Urbanism and Land Planning, University of Cantabria, 39005 Santander, Spain; valentin.castillo@unican.es; 2 Research Group of Health Economics and Health Services Management–Research Institute Marqués de Valdecilla (IDIVAL), 39011 Santander, Spain; david.cantarero@unican.es; 3 Department of Economics, University of Cantabria, 39005 Santander, Spain

**Keywords:** spatial patterns, COVID-19, microdata, geographic information technologies, ArcGIS, public health, geoprevention

## Abstract

Several studies on spatial patterns of COVID-19 show huge differences depending on the country or region under study, although there is some agreement that socioeconomic factors affect these phenomena. The aim of this paper is to increase the knowledge of the socio-spatial behavior of coronavirus and implementing a geospatial methodology and digital system called SITAR (Fast Action Territorial Information System, by its Spanish acronym). We analyze as a study case a region of Spain called Cantabria, geocoding a daily series of microdata coronavirus records provided by the health authorities (Government of Cantabria—Spain) with the permission of Medicines Ethics Committee from Cantabria (CEIm, June 2020). Geocoding allows us to provide a new point layer based on the microdata table that includes cases with a positive result in a COVID-19 test. Regarding general methodology, our research is based on Geographical Information Technologies using Environmental Systems Research Institute (ESRI) Technologies. This tool is a global reference for spatial COVID-19 research, probably due to the world-renowned COVID-19 dashboard implemented by the Johns Hopkins University team. In our analysis, we found that the spatial distribution of COVID-19 in urban locations presents a not random distribution with clustered patterns and density matters in the spread of the COVID-19 pandemic. As a result, large metropolitan areas or districts with a higher number of persons tightly linked together through economic, social, and commuting relationships are the most vulnerable to pandemic outbreaks, particularly in our case study. Furthermore, public health and geoprevention plans should avoid the idea of economic or territorial stigmatizations. We hold the idea that SITAR in particular and Geographic Information Technologies in general contribute to strategic spatial information and relevant results with a necessary multi-scalar perspective to control the pandemic.

## 1. Introduction

The globalization of the COVID-19 pandemic has led to massive health and socioeconomic problems worldwide. The COVID-19 continues its gallop across the world, testing the hospital capacity and strength of our health systems. This paper is addressed at a time when Spain, with a population close to 47.5 million inhabitants and after one of the most draconian COVID-19 lockdowns in Europe, has already exceeded 1.2 million COVID-19 cases, which implies a cumulative incidence of practically 521.07 cases per 100,000 inhabitants. In other words, an incidence rate more than 6 times the European average. Therefore, as long as coronavirus cases are rising, we can consider that a third wave is on the way and there is no much time to react now.

Furthermore, the Spanish case is relevant and important for two reasons: on the one hand, the collapse of the National Health Service due to full capacity of hospitalizations and Intensive Care Units, and on the other hand, the severity of the condition in the first wave of March 2020 and the difficulties from the multiple outbreaks since the beginning of the second wave in the summer period.

The dysfunction of the Spanish health service required strict confinement of their population for more than two months (called “State of Alarm”), which was followed by the subsequent de-escalated process based on three phases, which lasted for practically a month. In the new life after the end of the confinement, the misnamed “new normal” stage that began in the last week of June, there are teams of coronavirus trackers, health policy measures, and working teams (regional health ministries and central government). Unfortunately, the contact-tracing method in Spain is different from other countries, where policy-makers have considered using digital contact-tracing as a COVID-19 containment strategy to improve the traditional contact-tracing efficiency. Similarly, big data platforms have also been applied for tracing contacts as other studies show [1,2].

Our research is located within the collaboration framework established by IDIVAL Valdecilla (Department of Health of the Regional Government of Cantabria), where, among other issues, the SITAR (“Sistema de Información Territorial de Acción Rápida”, by its Spanish acronym) tool is presented as a Fast Action Territorial Information System that allows statistical, graphic, and cartographic analysis in real-time for certain hotspots.

Our study strongly supports the spatial perspective to face the spread of COVID-19 as a complement to the usual prevention measures based on health criteria and use of masks, hydroalcoholic gels, or the maintenance of a minimum distance of 1.5–2 m (6 feet) between people; however, buffer thresholds are still under investigation, and some recent works conclude the existence of airborne droplets from person to person at a distance greater than two meters [3] (p. 4).

Thus, to achieve our research objective, we implemented the SITAR data structure. SITAR, based on geotechnologies, is developed to face the sprawl of COVID-19 in the Autonomous Community of Cantabria. Moreover, this tool lets us analyze the cumulative spatial trend of COVID-19, disentangling when and, fundamentally, where another outbreak will occur at a local scale [4]. In this sense, it is important to highlight the strategic role played by geotechnologies to shorten response times for social management [5] and, more specifically, by SITAR to further the knowledge of spatio-temporal pandemic, overcoming the analysis scale of studies for all the country and the incidence of COVID-19 [6].

Therefore, if we consider the two stages for the risk mitigation, on the one hand, to reduce the spread and, on the other hand, to decrease the disease severity [7], this research contributes to the first one to reduce the spread, with local perspective (intra-urban scale). A geospatial and spatial-statistical analysis of the geographical dimension of the COVID-19 pandemic can be considered to understand the spatiotemporal dynamics of COVID-19 and the likelihood of new outbreaks. As Franch-Pardo et al. (2020) show, projection of spatial and temporal trends to face the challenges from an interdisciplinary perspective serves as a resource for understanding the evolution of tools used in the management of the major global pandemic of the 21 Century: the COVID-19 [8]. Moreover, COVID-19 studies with GIS and key determinants could be valuable tools in decision-making and health geographics from a social perspective in order to protect people. Following Franch-Pardo et al. (2020), our research line is related to two of five thematic groups of studies about geospatial analysis: spatiotemporal analysis and health and social geography [8]. Additionally, Franch-Pardo et al. (2020) establish three groups that exceed our goal: environmental variables, data mining, and Web-based mapping. Regarding the last one, outstanding and world-renowned is, in this sense, the role of the Johns Hopkins University scorecard, a clear reflection of the true digital revolution we are witnessing in the face of previous moments of geo-digital convergence [9,10].

Interestingly, health maps have always been an outstanding tool for tracking and combating diseases based on the analysis of the spatiotemporal evolution of new focus. However, it is the GIS Cloud technologies that in the COVID-19 case have an important role in providing the international community with dashboards and macro-modelers based on spatial intelligence principles [11,12] and advantages such as data integration coming from different organizations and the production of statistical and spatial information in real-time and accessible from anywhere in the world. More precisely, the mentioned initiative of the Hopkins team and the geotechnological developments of ESRI have been clear exponents. In this framework, the countries, depending on their data protection and treatment regulations, have opted for the application of geotechnologies in several levels and formats, even some cases in South Korea, to track and geolocate positive cases with good results in the management of the pandemic [13].

Some studies demonstrate that spatial patterns of COVID-19 present differences from one place and one time to another [14,15,16]. Hence, analyzing the cases with a temporal and multi-scalar perspective is important. For example, density is an important variable at a national or regional level but, by contrast, density is not the trigger or the main explanatory factor at a local scale. Moreover, the fact is that density is frequently associated with COVID-19, although it must be managed with limitations, being linked to other issues such as connectivity [14] or even referring to a density with conceptual details or limits, such as inhabited density [15]. Even, some research suggests the density as a key driver of an outbreak, but not with the severity or mortality [16].

On the other hand, at these scales of detail, differentiating social space issues are fundamental. This is demonstrated by several applied studies, which show that affection inequalities bring to the fore the social exposure to different determinants of health, due to the affection posed by the conditions in which people live, work, grow, and age [17] (p. 4). Moreover, in the analysis of vulnerability to COVID-19 carried out at the level of health areas, other papers identify a clear vulnerability derived from overcrowding and social content, as well as location, in the case of the latter referring to the economic orientation of several urban areas and finding a greater vulnerability in areas of important commercial activity [18]. Social vulnerability and the configuration of depressed areas into the cities increase the differences in the incidence of COVID-19, a pattern of socio-spatial affection that some studies even link to the ethnic or racial component, in that these populations tend to be located in socially vulnerable areas and eventually they end up being more affected by morbidity and mortality [19,20]. Similarly, income is other of the variables that positively correlates with the incidence of COVID-19, allowing to limit neighborhoods with the highest incidence on the intra-urban scale and to disentangle other social variables that could be decisive [21,22].

From a public health perspective, promoting and monitoring COVID-19 cases provides a meaningful strategy to improve healthcare outcomes. Geotechnologies, health maps, and spatial analysis give a brief overview of the key public health priorities, contributing to the geoprevention plan with a local and regional perspective.

## 2. Materials and Methods

The main source is the daily microdata record from the health authorities (Government of Cantabria—Spain), information managed only for research work by the Research with Medicines Ethics Committee from Cantabria (CEIm) in June 2020 (ID: 2020.238) [9]. In Spain, access to this kind of data is not common. Nowadays, there are only two Autonomous Communities, Galicia and Cantabria (of 17), and one province (Málaga) from another Autonomous Community where researchers are working with geotechnologies and COVID-19 microdata [23].

### 2.1. COVID-19 Daily Microdata Record

It is important to point out that microdata record presents an anonymous format due to the protection data laws requirements (not only at an international level: Regulation (EU) 2016/679 of the European Parliament and of the Council of 27 April 2016 but also at a national level: Organic Law 3/2018 Protection of Personal Data and Guarantee of Digital Rights of 5 December 2018). Furthermore, it should be noted that it is not an obstacle at all to ensure the research progress whether microdata register includes the variables as follows.

On the one hand, there are many fields related to the geographical location that ensures the geocoding process as: Address (street name and number), Locality (code and name), Municipality (code and name), and Postal code. On the other hand, the microdata register includes fields related to demographic characteristics, health structure, and virus details (Table 1).

Fields marked with * in the table above were included in this research because they are related to two characteristics of the COVID-19 pandemic in countries like Spain:

- The field Category indicates if the infected person works in the health sector. It is very important to consider the high incidence of COVID-19 in the health care providers (more than 55,000 infected, about 11% of COVID-19 patients in the country). This field enables to filter by the condition of health workers to avoid the distortion of results in the analysis stage.
- The field Elderly Homes is related to the high incidence of the first wave of COVID-19 in Spain in elder people who lived in retirement homes. In such cases, it is necessary to identify the location of elderly homes’ spatial focus (that commonly presents a high concentration of points in the same address). Otherwise, we could misread the statistical and cartographic results in the analysis stage.

Concerning microdata codification, it is important to consider the existence of a primary field to identify each person in the anonymous database, because the daily microdata file is accumulative. Therefore, the daily updating records is guaranteed by primary key joins.

Henceforth, we focus on other sources such as demographic data from census and municipal statistics (referred to spatial units: census sections), cadastral data from the Government of Spain (referred to buildings and parcels), location and dimension of sensitive public facilities (for instance hospitals, health centers, pharmacies, elderly homes or schools, among others). Aside from sources from official institutions (national or regional level), the research includes geographic, demographic, and socio-economic data from ESRI Spain COVID-19 GIS Hub, ArcGIS GeoEnrichment Service based on big data technologies and, finally, Web Map Services to connect to global cartography (satellite images, or similar).

### 2.2. A Methodology Based on Geographical Information Technologies Principles

This research is based on Geographical Information Technologies. More specifically, we use ESRI Technologies (a global reference in spatial COVID-19 research), in part due to the world-renowned COVID-19 dashboard implemented by the Johns Hopkins University team [9].

**Figure 1:**
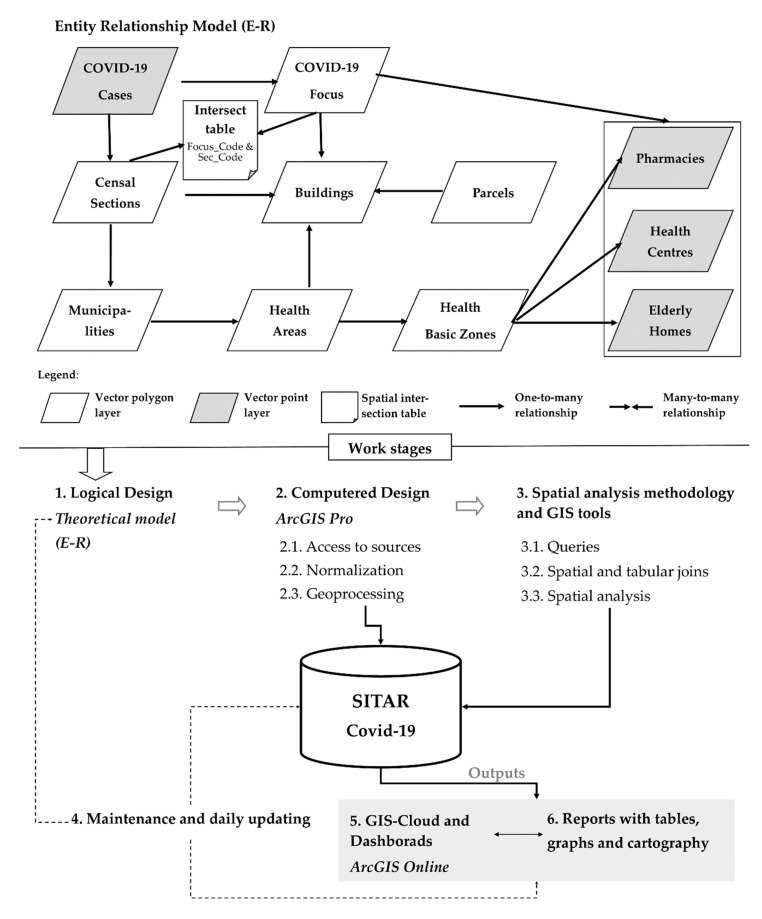
Work stages: from the logical design (Entity Relationship Model, E-R) to final products of SITAR (Sistema de Información Territorial de Acción Rápida).

In the ESRI tooling ecosystem, accessed by the use license from the University of Cantabria, we used ArcGIS Pro, as a desktop Geographic Information System (GIS) and ArcGIS Online, as a GIS-Cloud, with Operations Dashboard for ArcGIS, Web AppBuilder, and Experience Builder. Both GIS platforms, desktop and cloud, have been used to implement SITAR (Figure 1), a Fast Action Territorial Information System, to support with statistics and cartographic models a better knowledge of the COVID-19 evolution inside the region of Cantabria.

The working stages of SITAR are based not only on the life cycle of GIS projects but also on particularities for the daily updates of COVID-19 cases. Hence, a share of SITAR data is permanent during the research period but an important share of the data has to be daily updated and affects both geographic information components:

- Geographic component: location of infected people is daily updated due to a new case means a new point in the COVID-19 layer.
- Thematic component: the patient’s status can change, for instance, a positive case today can present a change of status tomorrow (a positive status can change into cured). The thematic updates affect to link layers (such as censal sections where number of positive cases is summarized or focus COVID-19 limits, among others).

The SITAR information is organized into three thematic geodatabases (GDB) that centralize and structure the data core of GIS tool created:

- GDB Health, which incorporates tiers of functional organization in health matters (health areas and basic health areas), both with polygon geometry, as well as fundamental facilities in relation to the main SITAR theme, such as the distribution of health centers, residences, and pharmacies.
- Socio-demographic GDB, which organizes tiers and basic background variables to identify the demographic and socio-economic determinants of the contagion sources. It is based on spatial units of sectioning, municipalities, and residential areas, all of them with polygon geometry.
- Cadastral GDB, which contains the tiers of parcels and buildings, with polygon geometry. This information is key for the detailed analysis of outbreaks. Furthermore, to overcome the absence of statistical data on the population at a more detailed level than the section, we point out that in this study the resident population at the building level has been modeled using GIS methods of downscaling [24].

**Figure 2:**
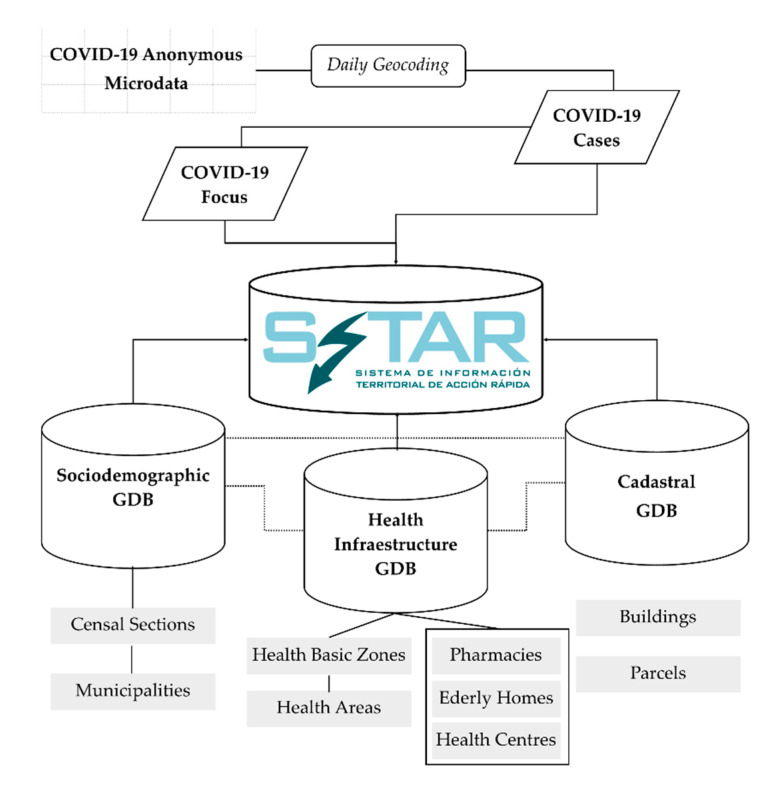
GIS data structure of SITAR in the computered design phase (geodatabases GDB).

In this conceptual GIS model (Figure 2), the COVID-19 case tier with initial point geometry takes on special prominence. On a daily basis and with a temporal perspective, it allows to analyze the evolution of spatial patterns and to define the COVID-19 hotspots at an intra-urban scale. As a result, from there is derived another of the basic information levels in this research, the focus.

The focus tiers are derived from COVID-19 cases (Active Filter) and correspond to a special polygonal unit that defines a problem area to be monitored or, where appropriate, on which the health authorities will act in a short term. In this regard, a methodological–conceptual precision must be made on the focus element in relation to the COVID-19 analysis. Although for health authorities the word focus refers to the point of origin or cause of a series of related infections, on the other hand, from the territorial point of view, in the GIS environment, focus is the special accumulation of active cases (which may be or not related to the same origin). Hence, two names are created that must be related and coordinated: health focus (linked to origin or causality) and special focus (related to the distribution of active cases).

The GIS data model implemented in the computered design phase of SITAR allows the spatial patterns of COVID-19 to be analyzed over time at detailed scales and to produce reports in real-time related to sources of contagion that may arise in any given moment.

**Figure 3:**
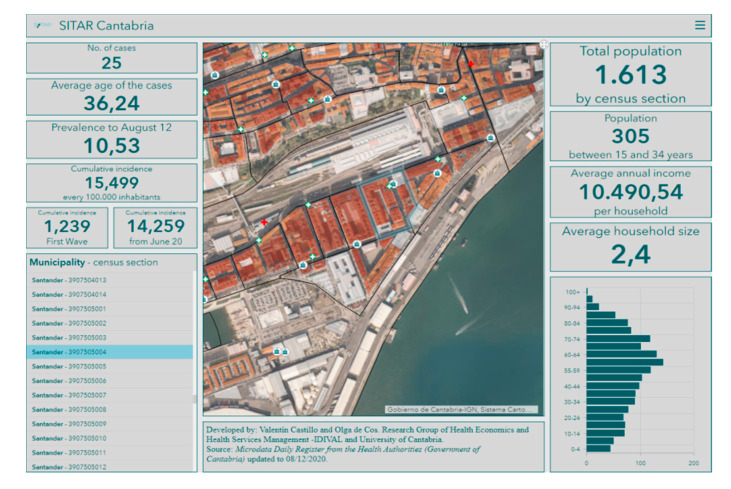
SITAR dashboard shows graphic and cartographic information about a censal section (selected polygon) in the city of Santander.

Likewise, as noted above, SITAR has been developed with ESRI GIS tools where there is a local data structure designed using ArcGIS Pro, while several outputs are developed for authorities through ArGIS Online. In this Cloud format, SITAR as a tool feeds an internal management scorecard that provides basic and complementary information to help decision-making at any stage of the COVID-19 pandemic. As mentioned above, GIS Cloud technologies have an important role in the COVID-19 management, especially the dashboards (Figure 3) that guarantee access to strategic information to design geoprevention plans. Moreover, it is an open and upgradeable tool that does not require any complementary software.

## 3. Results

Spatial analysis and queries from SITAR show several multiscale spatial patterns. This section starts with the current geographical setting of the Autonomous Community of Cantabria, as a case study, and only the most relevant results are presented based on the relationship between population, territory, and COVID-19.

### 3.1. Introduction: The Case Study

The Autonomous Community of Cantabria, located in the north of Spain, has just over 580,000 inhabitants and a surface area of 5300 km^2^ (2055 square miles), which represents an average regional density close to 110 inhabitants/km^2^ (285 inhabitants/squared meters). However, the distribution of the population and, therefore, the density present important internal contrasts. The capital city is Santander that it is located in the central coastal area with 172,000 inhabitants, around which a metropolitan area of dispersed growth is organized and supported by other smaller cities with high commuting in a metropolitan unit of 9 municipalities (of the 102 of the region) that concentrate about half of the total population.

**Figure 4:**
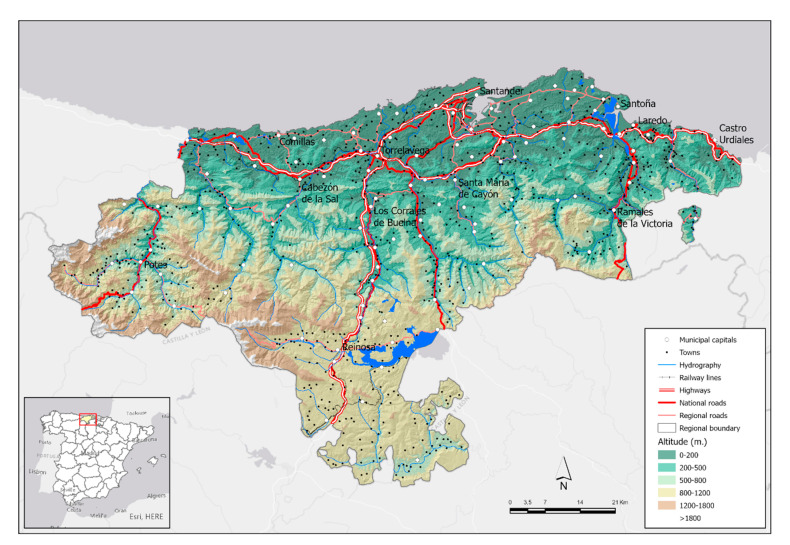
The territorial frame of Cantabria in the national context: orography, communication routes, and population centers. Source: Authors’ elaboration based on ESRI (Administrative Base map), National Geographic Institute (National Cartographic Base 200), and Government of Cantabria (Digital Elevation Model).

The second most important city is Torrelavega, an industrial-oriented one located 25 km (0.6 Mi) from Santander, with 51,000 inhabitants. The proximity of both cities and the reduction of the distance-time due to their direct communication by highway mean that they are interpreted as a polynuclear urban-metropolitan system. In fact, the Copernicus from the European Union Program has identified a single Functional Urban Area (FUA) in Cantabria that integrates the Santander-Torrelavega complex as a dynamic area that integrates urban systems with more than 50,000 inhabitants, in this case polynuclear, and with a prominent role in daily pendulum movements (commuting) between central areas and the surroundings [25]. Thus, an FUA made up of 25 municipalities is recognized with 380,000 inhabitants (just over 65% of the population of Cantabria). Density and mobility, territorial features frequently related to the presence of COVID-19 cases are two facts of this central sector of Cantabria (Figure 4).

Cantabria has a clear coast-inland duality in terms of population density. In addition to the FUA Santander-Torrelavega, the eastern coastal area stands out, influenced by the residential growth of the neighboring community, the Basque Country.

Otherwise, the region inside is organized around characteristic valleys, with low densities and population levels, but with an economic orientation to the primary sector, except in head towns, that allows a low spread of COVID-19, although present as a negative feature the rural tourism, which has led to a significant number of visits from other cities of Cantabria and the rest of Spain.

### 3.2. COVID-19 Spatial Patterns: An Approach to a Regional Scale

Since the pandemic began, nearly 12,000 cases have been registered in Cantabria and at the moment, the prevalence is about 2300 cases. This fact is growing daily with an average of 250 new cases registered per day.

On the basis of urban hierarchies by the Development Ministry of the Government of Spain [26] (Table 2), the higher concentration of COVID-19 cases in large urban areas (Santander and Torrelavega) is logical since both together house almost 40% of the population. Along all the period considered in these municipalities, slightly more than 44% of the cases happen, although the most outstanding process when isolating the cases in two periods (first wave and new normality stage with the second wave) is the higher concentration of cases in the municipalities of greater size, which exceed 51% of cases from the beginning of the new normal until the beginning of August. This increase of almost 9% compared to the first wave in the relative weight of COVID-19 cases in municipalities with more than 50,000 inhabitants also means a reduction in the relative accumulation of cases in the other hierarchies of municipalities: medium and small urban areas that reduce their relative weight in concentration by about 5%.

**Figure 5:**
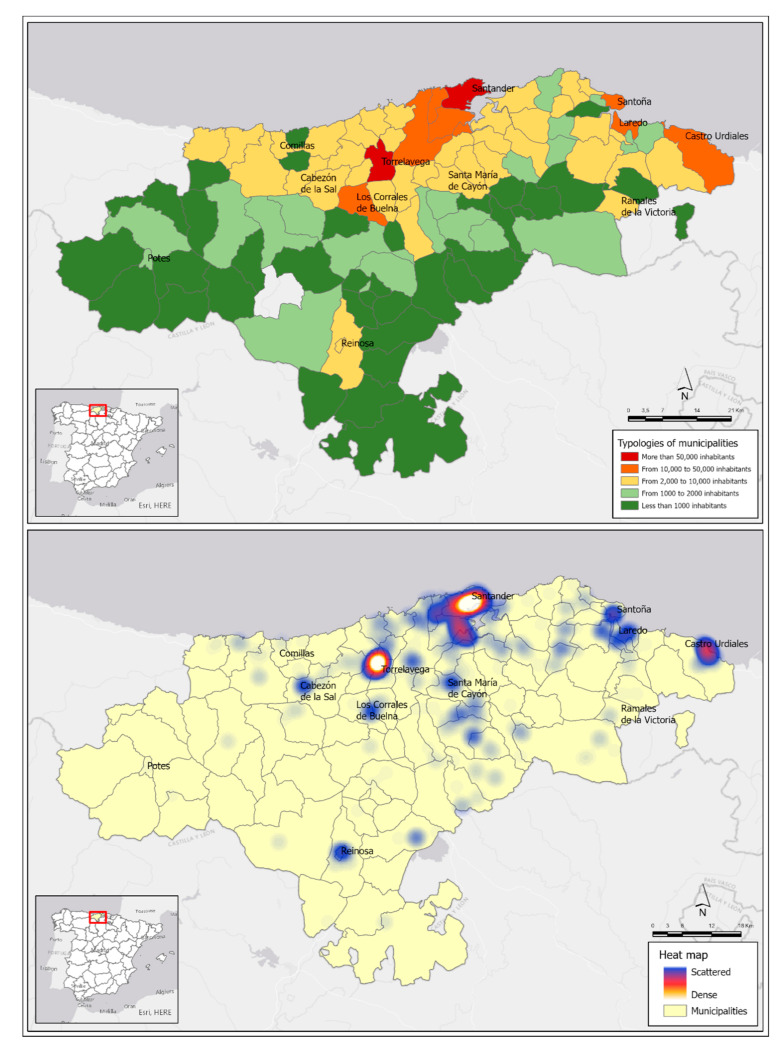
Regional spatial patterns. a. Territorial typology related to Table 1 by population hierarchy, b. Hot map of positive cases COVID-19 (Updated 25/09/2020); Source: Authors’ elaboration based on COVID-19 Microdata Daily Register from the Health Authorities (Government of Cantabria, Autonomous Community of Cantabria, Spain) and Municipal Census from the National Statistics Institute.

August was characterized by a contained trend of COVID-19 cases in Cantabria and it has meant that this region receives an important number of visits during the holiday period, which has had a direct effect on the new cases everyday registration, which has multiplied by 10 in the last month. Cantabria’s territorial organization, with this duality of a high-density coast and less populated interior valleys, is also reflected in the spatial patterns of the COVID-19 at a regional level (Figure 5).

**Figure 6:**
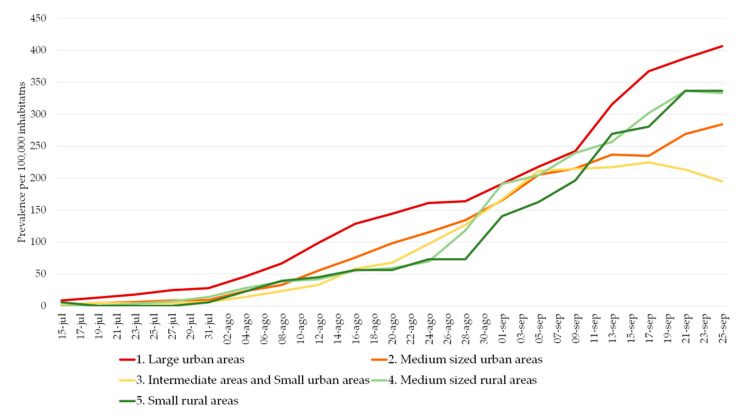
Evolution of prevalence per 100,000 inhabitants in Cantabria by territorial typology. Source: Authors’ elaboration based on COVID-19 Microdata Daily Register from the Health Authorities (Government of Cantabria, Autonomous Community of Cantabria, Spain). Software: ArcGIS Pro, Microsoft Access, and Microsoft Excel; Convergence of prevalence levels: in the last days, the range of prevalence values in opposite hierarchies (difference between red and dark green lines) represents only 50 cases per 100,000 inhabitants, while two weeks before the range represented the difference was double.

However, in all levels of government, the number of active cases is increasing. There is a generalized upward trend since the beginning of August, after a first stable stage at low prevalence levels (Figure 6). At the same time, the trend is convergent between larger and smaller municipalities.

### 3.3. COVID-19 Spatial Patterns: An Approach to an Intra-Urban Scale

The intra-urban scale is the most important one to reveal strategic spatial patterns. More specifically, it promotes risk mitigation. The analysis of the COVID-19 spatial patterns on an intra-urban scale has as a starting point the statistical verification of the distribution significance with a non-random pattern. It is applied to the two municipalities located in large urban areas, Santander and Torrelavega, that accumulate the greatest number of cases, the spatial average nearest neighbor, which measures the distance between each COVID-19 case and the location of the nearest neighboring one.

At an intra-urban level, the analyses carried out show a significant clustered distribution (Table 3), which is non-random and leaves a probability of less than 1% that the distribution of COVID-19 cases at an intra-urban level could be random.

In the spatial pattern statistical analysis, the null hypothesis is the complex aleatory pattern (CSR) and confirmation or rejection depends on the Z score (standard deviation) and *p* values (probabilities).

- The Z score is in both cases under the threshold value −2.58; therefore, the spatial pattern is clustered and not aleatory with a confidence greater than 99%.
- Small figures imply a low probability of aleatory spatial patterns; therefore, the null hypothesis should be rejected. In both study cases, Santander and Torrelavega, *p* = 0 so the distributions are not aleatory.

The average distance between a COVID-19 case location and the nearest neighbor is very short (about 18 m -59 ft- in Santander and 17 m -56 ft- in Torrelavega). Those values are far from the theoretically expected value (about 70 m -229 ft- in Santander and 77 m -252 ft- in Torrelavega).

The Ratio Nearest Neighbor presents values under 1 in both study cases; therefore, their spatial pattern is clustering (Santander 0.26 and Torrelavega 0.22).

From a geographical view, our results are fundamental to support the following analyses. The fact that the distribution is statistically significant shows a special structure (in this case clustering), allowing a solid deepening in the analysis of environmental variables and underlying socio-demographic content that shed light on how the coronavirus behaves spatially at detail scales.

#### 3.3.1. An Intra-Urban Pattern of COVID-19 Condition Linked to Social Content and Household Size

Based on the Franch-Pardo et al. (2020) classification of COVID-19 and geospatial research, the section will be framed by health and social geography studies [8]. Through spatial sampling for the COVID-19 cases, the SITAR tool provides several socio-economic and demographic variables in relation to the background in which the different COVID-19 cases are located. Isolating the municipalities cases of Santander and Torrelavega, the social content is analyzed on the variable of average annual income per person.

In both case studies, a greater concentration of cases is detected in areas with lower income levels. For example, Santander’s average income level is 11,359 euros and it should be noted that more than 65% of the COVID-19 cases located since the beginning of the pandemic are in districts with income levels below the municipal average.

The same socio-spatial pattern is highlighted in Torrelavega, with an average income of 10,815 euros and a distribution of 60% of COVID-19 cases in areas below the municipal average income level. Another variable shows a clear relationship with the concentration of COVID-19 cases and it points out the average size of the cohabitation units, like households. In urban centers there are a higher proportion of single-person households and this issue together with the high social content generates small oases in cases analyzed where the spread of the coronavirus is less important in contrast to the case concentration where the household size is greater.

**Figure 7:**
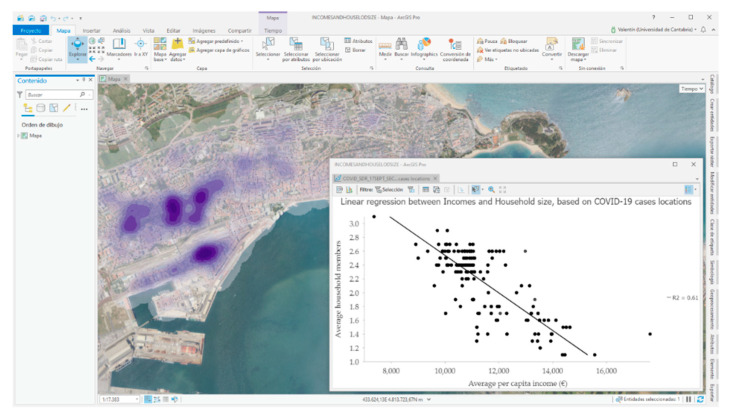
Working image SITAR: COVID-19 Kernel density model and linear regression between incomes and household size. Source: Authors’ elaboration based on COVID-19 Microdata Daily Register from the Health Authorities (Government of Cantabria, Autonomous Community of Cantabria, Spain).

Isolation of both variables (income and household size) for each case was done, where income is an independent variable with a determination coefficient of 0.62 (Pearson’s correlation coefficient of −0.78). In fact, a good share of the COVID-19 cases in Santander is distributed in districts with average annual income per capita levels between 10,000 and 11,000 euros in which there is also an average household size greater than 2.2 members (Figure 7).

#### 3.3.2. Spatial–Temporal Perspective: A Pattern of Spatial Repetition

SITAR analyses the case distribution with a temporal perspective, or more precisely with a spatial–temporal one. This is possible both from cartographic techniques, such as the expressive positive date-animated maps and more especially from thematic consultations and spatial analysis.

In this second approach, the microdata series of infected persons is organized into 2 data series:

- The cases of the first period, which extend from the start of the records in March until the start of the new normality stage after the confinement, a period which extends until 31 July 2020 with daily case records showing a coronavirus controlled evolution.
- The cases since 1 August, when the beginning of a second wave of the virus is already being identified in Spain and, more precisely, the coronavirus is spreading faster than in any neighboring nation.

An analysis of Euclidean distance is carried out based on the cases of the first period and the distance at which the cases of the second wave are located with respect to the closest cases of the first period. It is based on the calculated concentric distances. The results clearly show that the new cases produced closely repeat the special pattern of the previous ones. Hence, the new locations of COVID-19 cases are very close to positions where there were already some cases in the first stage.

**Figure 8:**
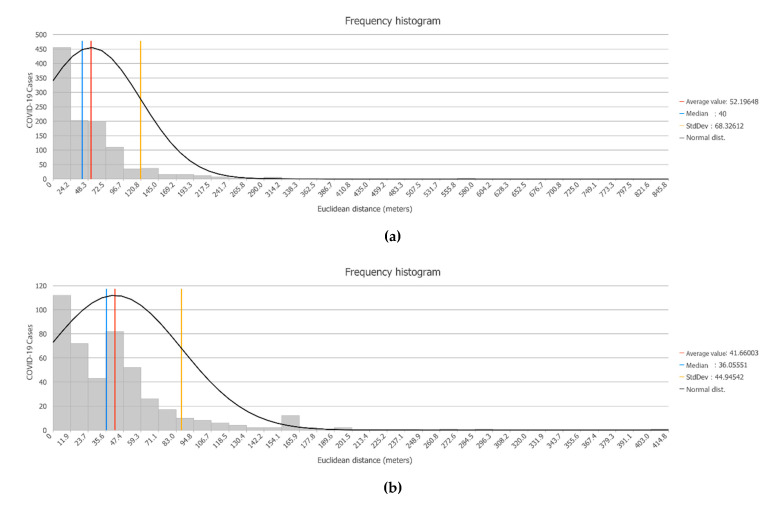
Relative distribution of new cases related to cases before (**a**) Distribution of new cases in Santander, (**b**) Distribution of new cases in Torrelavega. Source: Authors’ elaboration based on COVID-19 Microdata Daily Register from the Health Authorities (Government of Cantabria, Autonomous Community of Cantabria, Spain).

On average, the COVID-19 cases that have been located since August are about 50 m (164 ft) away from some cases in the first stage. In other words, the incidence area happens again, with varying intensity, resulting in different focus, but with similar intra-urban distribution patterns (Figure 8a). In fact, the modal distance is less than 25 m (82 ft), which occurs in 40% of the new cases, and three-quarters of the new cases are located practically less than 70 m (230 ft) from some COVID-19 case in the previous stage. The same issue happens in Torrelavega city (Figure 8b), with a modal distance to previous cases of less than 10 m (33 ft) and an average distance of about 40 m (131 ft). In this regard, our analysis provides new insights into the reasons for the design of geoprevention strategies by health authorities.

## 4. Discussion

Our findings show interesting spatial patterns of COVID-19 related to density, socio-economic content, and temporal perspective. First of all, we proved using SITAR that COVID-19 cases present a nonrandom pattern: we could identify a clustered structure. On this basis, regarding density, at a national and regional level, we identified a clear concentration of COVID-19 cases in main urban areas. Hence, our results are similar to other ones [14,16], even located in other countries. These studies find that population density can affect the timing of outbreaks through higher connectedness of denser location. This literature finds that population density is positively associated with proxies of social distancing and negatively associated with the population age. Furthermore, they find the metropolitan population to be one of the most significant predictors of infection rates because larger metropolitan areas have higher infection and mortality rates. However, countries with higher densities have significantly lower virus-related mortality rates than countries with lower densities, possibly due to more resilient health care systems [16].

Additionally, from another point of view, the COVID-19 pandemic has several implications that clearly go beyond the health field, even calling into question the urbanization model that mega-cities have starred in worldwide, with a fitted population concentration pattern from an economic point of view. Nevertheless, in the current situation, they pose a clear risk due to their favoring effect on the transmission of the coronavirus, something that has been evidenced as a problem and that may also be very similar in the future for the new virus [27,28].

Regarding the relation between COVID-19 and socio-economic profile [19,20,21,22], our study reveals that there is the highest case concentration in areas with low income levels (up to 11,000 euros per household per year) and with a larger average size (mainly from 2 people per household). Thus, a much laxer behavior of cases are observed in sections with higher income levels and reduced average household sizes. In other words, the literature points out important differences in the proportion of the spread of the COVID-19 depending on socio-economic patterns and household size and they are very similar to the differences in the spatial evolution of the COVID-19 pandemic in our study.

Moreover, our findings show that if we focus on the usual pattern described (sections with less than 11,000 euros of average per capita income and households with more than 2 members), we observe that more than 65% of COVID-19 cases are located in areas with these characteristics. In this regard, it should be noted that this distribution partly responds to the distribution pattern of the total population in the region. Hence, if massive testing were chosen from a geoprevention perspective, addressing the resident population of these sample sections would lead to sample 295 census sections in the region (63% of the sections) with a total resident population of close to 372,000 inhabitants.

A novel contribution of our research is the demonstration of the value of micro-scale sources (microdata) to analyze the spatial behavior of the virus with a multi-scalar perspective. In order to generate valid knowledge on geoprevention, it is basic to start from individual data and aggregate them into focus [23], neighborhoods, etc. Based on this approach, our study provides a valid methodology that could be used by other research teams and they could be applied to other countries (including an adaptation, if necessary) with the only requirements that their authorities have individualized COVID-19 cases checking and the research group gets permission to use the COVID-19 microdata. In this regard, a weakness refers to an unequal availability of statistical sources of a demographic and social nature in countries in which the SITAR methodology is applied. In fact, with similar geo-demographic analysis in relation to COVID-19, it is possible to find studies in which the reference census sources are significantly obsolete. In other words, 8 or 10 years ago is a weak variable for a better understanding of the most dynamic areas of cities and this would lead to discuss the results achieved. Therefore, it is very important to have updated socio-demographic sources, since in several case studies the COVID-19 data are related to the demographic and social census data, in most cases ten-year and often leading to comparison with scenarios.

In relation to our methodology, we hold the idea if the COVID-19 is a multi-scalar problem, a clear conclusion is that this technical analysis is useful. Another shortfall is derived from the problems of new groups with weight and presence in the media and the risk of negative groups and the impact they could have. This is related to one of the elements that increase or mitigate the COVID-19 risk perception trust due to people or organizations that dispute the adequacy and effectiveness of the coronavirus measures [29].

Nevertheless, our study has several limitations beginning with the use of SITAR tool in geoprevention. First, related to the GIS challenge established by KAMEL [4] and successful experiences based on tracking [13], it would be essential to have the geolocation of the identified contacts by the trackers because for 14 days, apart from new cases, these locations point to places where new positives could be produced by the effect of the infections chain. A possible improvement could be the fact that the Spanish Institute of Statistics is working on new mobility variables at the district level, and we raise that it will be very useful for tools such as SITAR.

Future research should consider that coronavirus is hitting household health and incomes hard, something that has not been mentioned so far. Moreover, we know the population that lives in a region or a country but how many living units do affect the spread of the coronavirus is a question pending to solve. At least we can get to narrow it down to the building level but we do not know if all of us live in cities with the same composition or it is related to density, overcrowding, or something related to social content.

On the other hand, there is another research line with a predictive orientation, usually national or at most regional in scope, and all this despite the fact that these studies frequently point out the difficulty of determining where the virus is going to spread from the first weeks of incidence. However, modeling can help to manage and mitigate the pandemic, especially in the development of preventive measures [30,31].

Lastly, it would be advisable to compare regional cases with others of countries that share similar demographic or clinical characteristics.

## 5. Conclusions

Our findings demonstrate that the existence of a nonrandom and grouped geographical or spatial pattern is verified, which opens up great opportunities for advances in geoprevention based on the analysis of the spatial behavior of the coronavirus. Additionally, our study highlights that density matters in the spread of the COVID-19 pandemic at a regional level. Large metropolitan areas, functional urban areas, or district with a higher number of persons tightly linked together through economic, social, and commuting relationships are the most vulnerable to pandemic outbreaks being more especially in countries like Spain or their regions. Nevertheless, at an intra-urban scale, the socio-economic content is more determinant than density related to COVID-19 distribution. Hence, it is important to point out that in the theories relating to the characterization of COVID-19, the scale is fundamental and in that a valid statement at the country, provincial, or department level can fade or, on the contrary, be strengthened, at a scale of detail with an intra-urban perspective (at the neighborhood level). Therefore, we understand that the knowledge of the special behavior of COVID-19 requires a multi-scalar analysis [29,32]. Within the intra-urban scale, the spatial analysis can produce revealing knowledge to help policymakers to reduce the COVID-19 spread, aiming one of the stages of the risk mitigation [7].

SITAR, based on geographic technologies and digital twins philosophy, demonstrate that it is very useful to include geotechnologies to produce strategic information, and society, neighbors, and civil action should be involved even before a lockdown. Considering the important role of GIS Cloud Technologies, SITAR dashboard is a new contribution to facilitate the access to COVID-19 spatial information not only to policymakers but also to society. Related to this, a geographic cloud viewer that could be consulted from local administrations in real-time, centralized by regional governments to support prevention, can be a good proposal for the next future to provide new insights in relation to the spatial behavior of the virus. It is essential to combine disciplinary knowledge (health and social geography research lines) with geotechnologies to contribute with new interdisciplinary knowledge adapted to the pandemic issue. All this generates relevant knowledge for the design of control and prevention strategies by health authorities; prevention with a territorial perspective, which is an evocative term leads us to consider geoprevention in health matters.

Moreover, at this moment, we have demonstrated that the distribution of COVID-19 shows a special structure (in this case clustering), allowing a solid deepening in the study of context variables and underlying socio-demographic content that shed light on how the coronavirus behaves spatially at detailed scales. Finally, we hold the idea, the importance of microdata, geocodification, and geotechnologies to know the behavior of the COVID-19 from a complementary perspective: the spatial one.

## Figures and Tables

**Table 1:** Microdata variables related to demographic and health characteristics.

Topic	Variable	Format: Values
Demographic structures	Sex	Text: Man/Woman
	Age	Number
	Category * (only health workers)	Text: Nurse, auxiliary nurse, or emergency physician, among others
Health structure and virus details	Start date	dd/mm/yy
	End date	dd/mm/yy
	Health area code	Text: five characters official code
	Health area name	Text
	Status	Text: Positive (Active Virus), Cured, Deceased
	Deceased date	dd/mm/yy
	Hospitalized	Binary text
	Intensive Care Unit	Binary text
	Hospital Name	Text: Hospital Acronymous
	Test Type	Text: ITC, PCR, QLIA, Rapid Test
	Elderly Homes *	Binary text

**Table 2:** Positive COVID-19 cases by territorial typology. First period of the new normality phase.

Territorial Typology	Map	Population 2020	Total Positive Case 25/09/2020	Prevalence Per 100,000 Inhab. 25/09/2020	14-Day Cumulative Incidence per 100,000 Inhab. ^1^
Large urban areas (Municipalities >50,000 habitants) ^2^		224,033	911	543	269
Medium-sized urban areas (Municipalities from 10,000 to 50,000 habitants) ^3^		151,984	432	327	167
Intermediate areas and Small urban areas (Municipalities from 2000 to 10,000 habitants)		158,434	309	340	124
Medium-sized rural areas (Municipalities from 1000 to 2000 habitants)		28,810	96	69	238
Small rural areas (Municipalities <1000 habitants)		17,817	60	35	289
Total Cantabria		581,078	1160	1314	202

Source: Authors’ elaboration based on COVID-19 Microdata Daily Register from the Health Authorities (Government of Cantabria, Spain) and Municipal Census from the National Statistics Institute. ^1^ Reported positive cases between 11/09/2020 and 25/09/2020 per 100,000 inhabitants. ^2^ This population interval corresponds to Santander y Torrelavega municipalities. ^3^ It includes several municipalities, such as (in population volume decreasing order): Castro-Urdiales, Camargo, Piélagos, El Astillero, Santa Cruz de Bezana, Laredo, Santoña y Los Corrales de Buelna. Note: calculated fields in Table 1 are referred to COVID-19 cases where the microdata register identifies the location (address to geocodify). Otherwise, the cases are accounted for as a separate category called “not located”.

**Table 3:** The spatial average nearest neighbor: Santander and Torrelavega cases.

Statistical Results	Santander	Torrelavega
Observed distance	17.9 m	17.0
Expected distance	69.5 m	76.5 m
Ratio nearest neighbor	0.26	0.22
Z Score	−62.3	−46.2
*p* value	0.00	0.00
**Graphical results** **Santander**	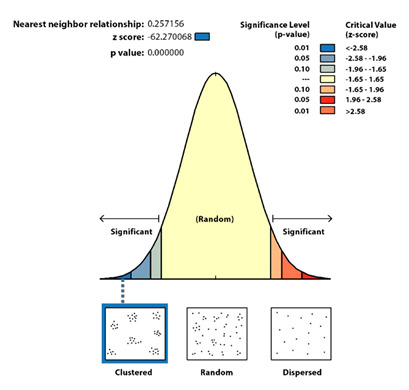	
**Graphical results** **Torrelavega**	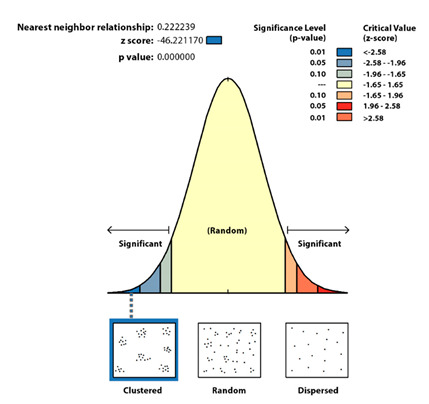	

Source: Authors’ elaboration based on COVID-19 Microdata Daily Register from the Health Authorities (Government of Cantabria, Autonomous Community of Cantabria, Spain).
